# Reliability of a New Test of Balance Function in Healthy and Concussion Populations

**DOI:** 10.3390/jfmk5010013

**Published:** 2020-02-14

**Authors:** Mihaly Kis

**Affiliations:** Department of Neurosurgery, University of Toronto; Toronto, ON M5T 1P5, Canada; mihaly.kis@thp.ca

**Keywords:** concussion, mTBI, balance, balance error scoring system

## Abstract

Providing quantitative measures of balance and posture is a valuable aid in clinical assessment and in recent years several devices have been introduced that have demonstrated the accurate measure of balance via deviation of center of mass utilizing software algorithms and mobile devices. The purpose of this study was to assess the accuracy of EQ Balance against the Sway^TM^ Balance System (Sway), another balance device that is currently established as an accurate measure of balance, and to evaluate the test–retest reliability of EQ Balance. Seventy individuals presenting to a sports medicine and concussion clinic volunteered to participate in the assessment of balance utilizing Sway and EQ Balance simultaneously. The group included 25 males and 45 females (mean age: 37.8 ± 14.8, range: 13–65) with and without concussion or other neurological conditions (39 concussed vs. 31 non-neurologically injured, or healthy). Twenty-six of the concussed participants were balance-impaired. Participants performed five postures while holding the mobile device against their chest. Participants held a device holder that secured two devices: one iPhone 6 with EQ Balance and a second iPhone 6 with Sway Balance. The average balance score on all five stances was recorded as the “average balance score”. Average balance scores were in statistical agreement between the two methods across the entire group, and for sub-groups according to the Deming regression (*p* < 0.01). The intra-class correlation (ICC) for the cohort was 0.87 (*p* < 0.001). Across the cohort, EQ Balance measured significantly worse balance scores in the balance-impaired group, comprised of participants with brain injury who failed a clinical balance screening test, compared to the group without clinically-determined balance impairment (this group includes healthy and some concussed patients). EQ Balance demonstrated safety, as it was considered safe to perform independently (i.e., without an observer) in those with impaired balance, and high test- retest reliability in the healthy and concussed patient population. Statistical agreement was established between the two measures of EQ Balance and Sway Balance for the average balance score across all five stances. The ICC analysis demonstrates strong consistency of the task output between test sessions. Given these results, EQ Balance demonstrates strength as a new balance assessment tool to accurately measure balance performance as part of a unique and novel gamified application in healthy and neurologically injured populations.

## 1. Introduction

Balance is a term that describes the integration of sensory, motor, and bio-mechanical processes that enable a person to maintain their center of mass within their base of support [1,2]. Maintenance of balance, or postural stability, is a critical component of motor skills and ranges from the simple maintenance of posture to the performance of complex voluntary movements in athletics or daily living [3,4,5]. Clinically, improvement in balance function has been shown to support injury prevention, promote recovery from injury, and facilitate improved functional athletic performance [3,6,7]. Given the preceding, the quick, objective and reliable assessment of balance for all populations, in any clinical, athletic, recreational, or wellness setting, is important.

Subjective and objective methods of balance are commonly used in clinical practice [8,9,10]. Subjective tests usually include a set of testing and scoring procedures; these vary widely across tests and tend to be restricted to clinical settings because they rely on the knowledge and experience of the test administrator [11]. These subjective balance tests have the benefit of being easy to administer and requiring minimal equipment [10]. However, most do not provide quantitative data and, furthermore, they rely on the skill and experience of the test administrator for scoring and interpretation, limiting the ability to compare across multiple test sessions and/or multiple sites [12,13].

There are also a number of objective methods of balance assessment. These include the use of devices, such as force platforms, strain gauges, and accelerometers in mobile phones and tablets [14,15,16,17]. Recent studies have evaluated the use of cost-effective technologies, such as wearable accelerometer-based devices and video-game technology, against sophisticated devices for gait and balance assessment [18]. Additionally, wearable sensors can provide valuable information on joint position and biomechanic metrics of movement [19]. These various digital devices generate quantitative scores by which clinicians and researchers are able to track change over multiple tests [20,21]. Some of these devices, while accurate, require large and difficult to transport equipment and, as such, are generally limited to institutional and laboratory assessment. In contrast, accelerometers contained in mobile phones, and tablets are small, lightweight, and able to be attached to the subject. Moreover, they have become commonplace and readily accessible in most if not all segments of society [22]. Two FDA-approved implementations of wireless accelerometer systems that are in use today for quantifying the attributes of gait and balance are the Sway Balance^TM^ (“Sway”) and K-D Balance systems [22] (510(k) Summaries: K-D Balance K173669, Sway K121590). 

Our study is focused on demonstrating the clinical equivalency of the balance assessment between EQ Balance, a gamified mobile device balance assessment application, and Sway, a more traditional mobile device balance assessment application. In addition, the study included a test–retest analysis intended to test EQ Balance’s intrasession reliability, or stability through time. In the context of this clinical study, test–retest variance included variability in the participants’ balance performance as well as measurement variability introduced by the device.

The purpose of this study was to assess the reliability and accuracy of EQ Balance and to compare its performance against another established software application that utilizes mobile devices to measure balance, Sway Medical.

## 2. Materials and Methods 

Performance testing was completed by Highmark Health Sport and Concussion Clinic. EQ Balance (Highmark Interactive, Toronto, Canada) and Sway (Sway Medical, Aledo, TX, USA) testing were performed for a total of 70 subjects (25 male, 45 female) with and without concussion or other musculoskeletal conditions (39 concussions vs. 31 non-neurologically injured, or “healthy”). A subgroup of the concussed participants had confirmed balance impairment, determined by failure of the modified Balance Error Scoring System (mBESS) test, as part of the standard testing protocol used by the study physician (26 participants were “balance-impaired”) [23]. As participants presented to HighMark Sport and Concussion clinic at various time points following concussion, the timing of administration of the mBESS test differed amongst participants. While the authors acknowledge the limits of the mBESS test to contribute to the diagnosis of concussion following the initial 3–5 days post injury, the mBESS was used in the study as a screening tool for balance deficits as opposed to a diagnostic tool for concussion.

The mBESS protocol requires participants to balance in three stances: double leg, single leg and tandem stance, with eyes closed and hands placed on hips, on a firm surface, for 10 s each [23]. The maximum balance score is 30, with points subtracted for each mistake with a maximum of 10 points subtracted per stance [23]. This score was used by the clinician to diagnose balance impairment in the context of the clinical visit, while taking into account the normative data for the patient’s demographic [24,25,26].

**Figure 1:**
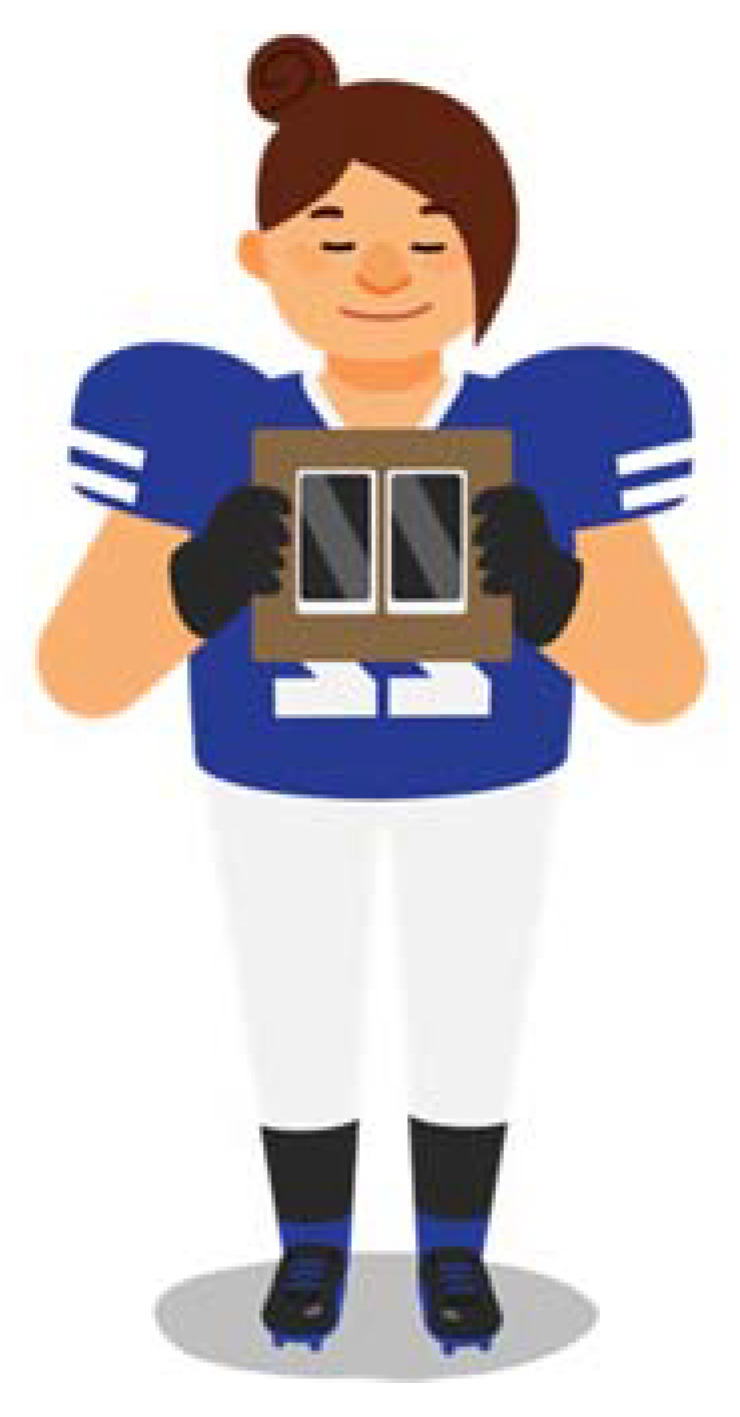
The two devices were affixed to the rigid device-holder and participants were instructed to hold the device-holder against their chest.

In the study trials, balance measures were obtained for a total of five stances, which included the double leg stance, right tandem stance (i.e., right foot behind), left tandem stance (i.e., left foot behind), right single leg stance and left single leg stance. Each stance was held for 10 s, completed on a firm surface and with eyes closed. The amount of movement was calculated by each balance test application algorithm using data generated by the mobile device accelerometer. A device holder secured two iPhones (version 6s, Apple, Cupertino, CA, USA), one running EQ Balance and the other running Sway. The devices were held by the participant at chest level against the approximate mid-point of their sternum, and the devices were confirmed to be in equivalent triaxial planes of movement. See Figure 1, below, for a depiction of how the devices were held. A total of three trials were completed. The first trial was considered the practice trial and was excluded from analysis. This was immediately followed by a second trial where EQ Balance and Sway were used simultaneously. Immediately following this, a third trial was conducted with EQ Balance alone, to collect reliable data. The EQ Balance and Sway applications were initiated and terminated simultaneously in order to maintain synchronized recording. Upon completion of the test, the scoring data generated by EQ Balance and Sway were documented for analysis. Descriptive analysis, Spearman’s rho, intraclass correlation, standard error of measurement (SEm) and all figures were completed with IBM SPSS Statistics Subscription 1.0.0.1072 (IBM, Armonk, NY, USA). Intraclass correlation coefficient (ICC) was used to calculate reliability. ICC was calculated with a two-way mixed effects model, evaluating for single measurements, and absolute agreement between tests [27]. SEm was calculated by multiplying the standard deviation of the scores in the group by the root of 1 − reliability, where Chronbach’s alpha was used for reliability. Deming regression was calculated using MedCalc Statistical Software version 19.0.4 (MedCalc Software bvba, Ostend, Belgium; https://www.medcalc.org/; 2019). To calculate Deming regression, coefficients of variation were calculated for each measure separately using test results from timepoint 1 (T1) and 2 (T2), based on within-subject standard deviation for each test. These coefficients were then used to calculate the Deming regression for measures at T2. The intercept and slope were calculated according to Cornbleet and Gochman [28]. Standard errors were calculated using the jackknife method described by Armitage et al. 2002. Standard errors were used to run a t-test to evaluate the difference between the Deming regression equation and y = x + 0. That is, a *t*-test was calculated for the slope and intercept separately, where the intercept was compared against 0 and the slope was compared against 1. This was conducted for each pose and each subgroup. For Deming regressions that included the entire cohort of 70 participants, the coefficient of variation was calculated on that dataset. However, for subgroups, the smaller number of participants made the coefficient of variation less reliable so the coefficient of variation was taken from the full cohort’s average of all poses.

### 2.1. Post-Hoc Analysis: Group Comparison

A post-hoc analysis was conducted to investigate the difference in balance performance, as measured with EQ Balance, between healthy and balance-impaired cohorts. A Mann–Whitney test was used as a non-parametric test to compare balance performance based on balance injury status (i.e., healthy vs. impaired balance). This analysis was completed with IBM SPSS Statistics Subscription 1.0.0.1072.

All testing procedures were approved by the Veritas Institutional Review Board for Research involving Human Subjects, and informed consent was obtained from all subjects prior to participation.

### 2.2. Instrumentation

The current study utilized the Sway Balance mobile application (Sway Medical, Aledo, TX, USA) and the EQ Balance application (Highmark Interactive, Toronto, Canada) installed on two Apple iPhones (version 6s, Apple, Cupertino, CA, USA). The Sway application, installed on the mobile device, accesses the output of the device’s accelerometer to determine a balance score. The units representing the balance score are determined by undisclosed calculations from Sway Medical. A validation study of Sway compared the output of Sway results to a static force plate (Biodex Balance System SD, Biodex Medical Systems, Shirley, NY, USA) while participants did a single-leg stance for 10 sec. The study found no significant difference between the two balance scores and a significant correlation between the two sets of results, across the cohort of 30 participants [22]. 

Similarly, EQ Balance application, installed on the mobile device, accesses the output generated from the accelerometers to determine a balance score. The units representing the balance score are also determined by undisclosed calculations from Highmark Interactive.

These mobile device systems incorporate accelerometers that measure the instantaneous acceleration of an object at any given point in time. These accelerometers, termed tri-axial or tri-axis accelerometers, incorporate three orthogonal measurement axes that quantify acceleration independently yet are housed in the same device. 

## 3. Results

### 3.1. Descriptive Analysis

In total, 70 participants were enrolled into the study. The entire study cohort was comprised of 39 individuals with concussion and 31 healthy controls. Demographic information is presented in Table 1 below.

**Figure 2:**
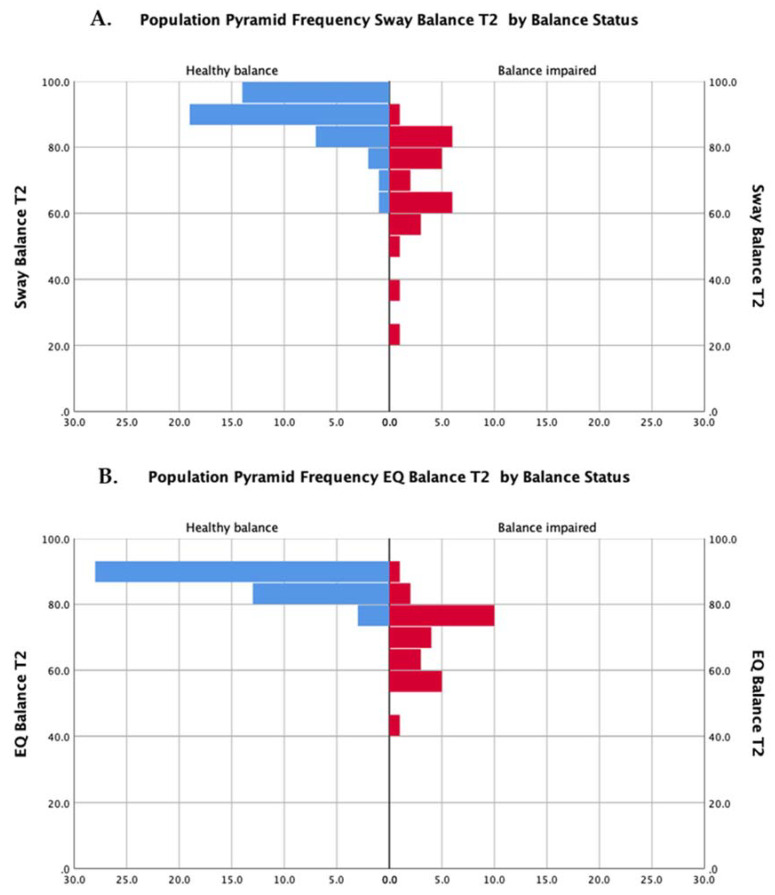
Distributions of scores for participants with balance impairment (red bars) and healthy balance (blue bars) for the Sway Balance task (**A**) and the EQ Balance task (**B**).

The distributions of scores for both methods of assessment (EQ Balance and Sway) are presented in Figure 2. 

**Figure 3:**
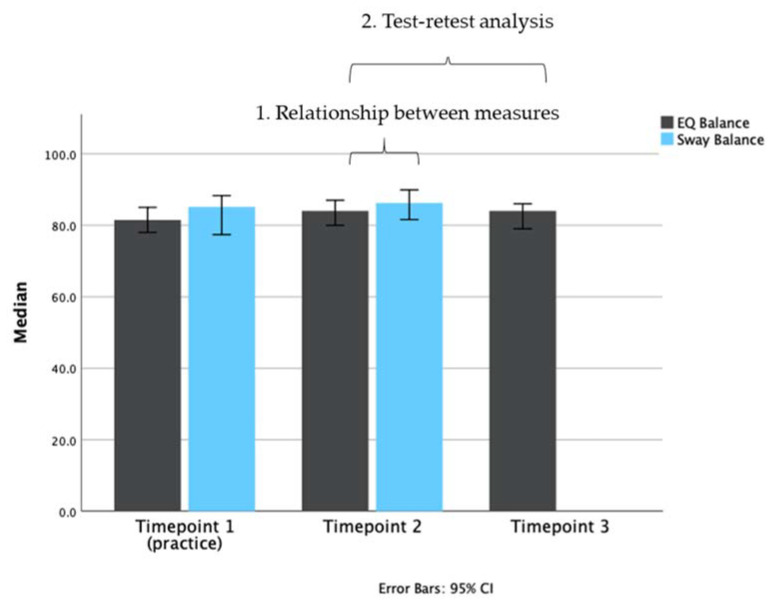
Bar plot representing the median scores of each test (EQ Balance, gray bars; Sway Balance, blue bars) at different time points. Timepoint 1 (T1) was used as a practice/acclimatization session. Timepoint 2 (T2) was the main test session. Timepoint 3 (T3) was used in the test-retest analysis for EQ Balance. Data are plotted as medians with 95% confidence intervals.

Means for the assessments conducted at each time point are presented in Figure 3.

### 3.2. Analysis 1: Relationship between Measures

Methods were compared using Spearman’s rank-order correlation to assess the strength and direction of association between measures, and a Deming regression to assess each pose individually. 

Deming regression was derived for validity assessment. Table 2 shows Spearman’s rho (rho) correlations and Deming regressions for results of each pose across all participants and two subgroups: healthy balance and impaired balance. Across all participants, a strong, statistically significant correlation was identified between the measures (rho = 0.848, *p* < 0.001). Separating by subgroup finds a strong correlation for all poses except for balance-impaired during right single leg stance where a weak correlation was found (rho = 0.43, *p* = 0.03) and in healthy participants with feet together, where there was a non-significant positive correlation between measures (rho = 0.21, *p* = 0.177). 

By observation, this analysis identifies tight clustering of individuals both with and without concussion whose balance deficits are relatively low (i.e., those with scores > 80.0). Greater dispersion is observed with those from both groups with apparent balance deficits; however, these data maintain a strong correlation: running a correlation analysis on the subgroup of participants with an EQ result below 80 found the Spearman’s correlation coefficient, rho = 0.86 (*p* < 0.001) between measures in this group.

**Figure 4:**
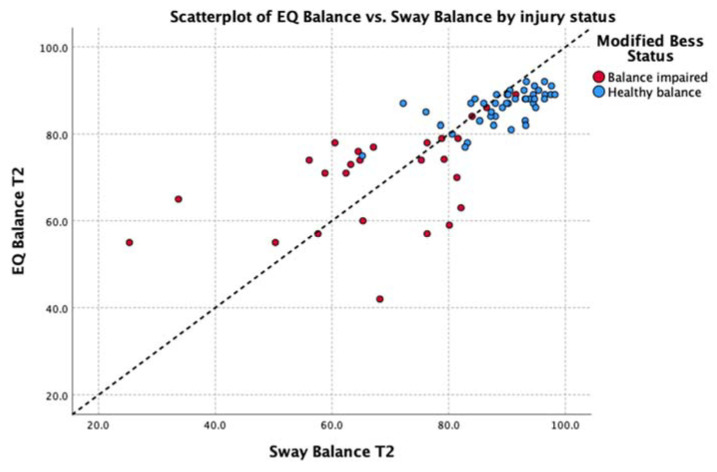
Relationship between the scores on the Sway Balance task and the EQ Balance task. Data points are differentially identified by balance impairment as measured by the mBESS (blue = pass, red = fail). Unity line Y = X is shown as a black dotted line.

Deming regressions were run to compare Sway and EQ while accounting for variability in both measures. This analysis of the average balance score across the entire group found EQ Balance = Sway Balance * 0.78 + 18.36, with the t-test finding no significant difference from y = x + 0 at *p* < 0.95%. Table 2 shows Deming regression results for EQ Balance vs. Sway Balance for all poses, where x represents the Sway Balance score and y represents the EQ Balance score for the given pose. Figure 4 illustrates the relationship between scores on tasks in comparison to unity (y = x).

The median values for healthy participants are shown, by pose, in Table 2. The Wilcoxon signed ranks test was used as a non-parametric method for calculating the difference between the two measures. It failed to find a statistically significant difference between the results of each test (z = −1.958, based on negative ranks, *p* > 0.05 2-tailed).

### 3.3. Analysis 2: Test-Retest Analysis of the EQ Balance Task

**Figure 5:**
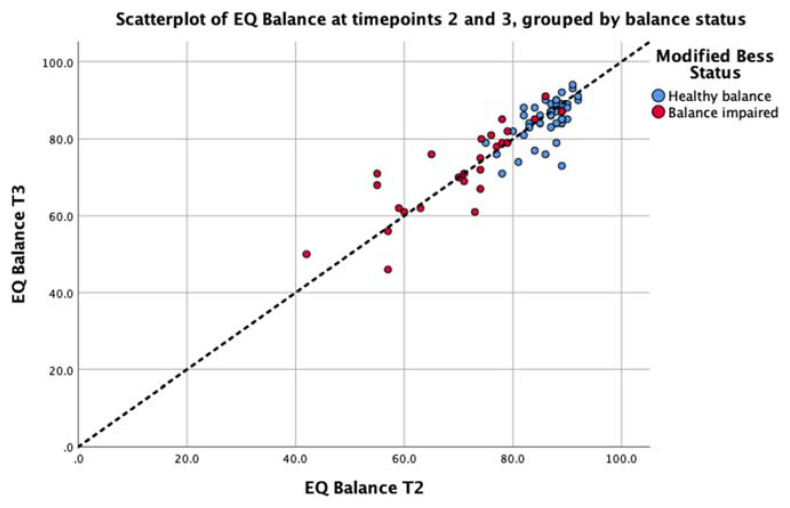
Scatterplot depicting the test–retest data for the EQ Balance task taken at Timepoint 2 (T2) and Timepoint 3 (T3) for the balance-impaired group (red) and healthy group (blue). The unity line (y = x) is represented by the dashed line.

Consistency of the EQ Balance measure was conducted by implementing the assessment on two successive occasions on the entire study cohort. The intra-class correlation for the cohort was 0.87 (*p* < 0.001) with a 95% confidence interval of 0.81 to 0.92. The standard error or measurement was calculated as 2.72. EQ Balance task scores for each test session are presented in Figure 5. This analysis demonstrates strong consistency of the task output between test sessions.

### 3.4. Post-Hoc Analysis: Group Comparison

As a post-hoc analysis, the Mann–Whitney test was used as a non-parametric test to identify a difference in balance performance, measured with EQ Balance, between healthy and balance-impaired cohorts. The results of this test for each measure, and each pose are shown in Table 3.

## 4. Discussion

Balance assessment tools can be utilized to assess the neuromuscular effects of aging, identify neurological disorders, aid in the diagnosis and management of injury related to brain trauma, and identify functional deficits related to activities of daily living. Additionally, assessment tools are critical in the successful implementation of a balance training program to demonstrate the current level of function and to track progress achieved over time. Measures of balance performance can provide valuable information on recommended training for injury prevention and improving athletic performance and, therefore, can be used as a prescriptive rehabilitative tool in injury recovery [4,5,13,29,30]. However, assessment protocols must be reliable, valid, reproducible, and sensitive enough to measure significant changes, all of which are common limitations to subjective clinical tests. 

Exploration of the dataset revealed that balance results, as measured by both tools, were negatively skewed (Figure 2). This is expected to occur on tests with an upper bound and where the clinical group under investigation can still be high functioning. Recent work by Inness and colleagues [31] identified that only 32–48% of adults with concussion demonstrate balance deficits on sway-related metrics. The distributions were comparable between tests (i.e., similar distributions in both EQ Balance and Sway) and between individuals (i.e., concussion and healthy controls).

**Figure 6:**
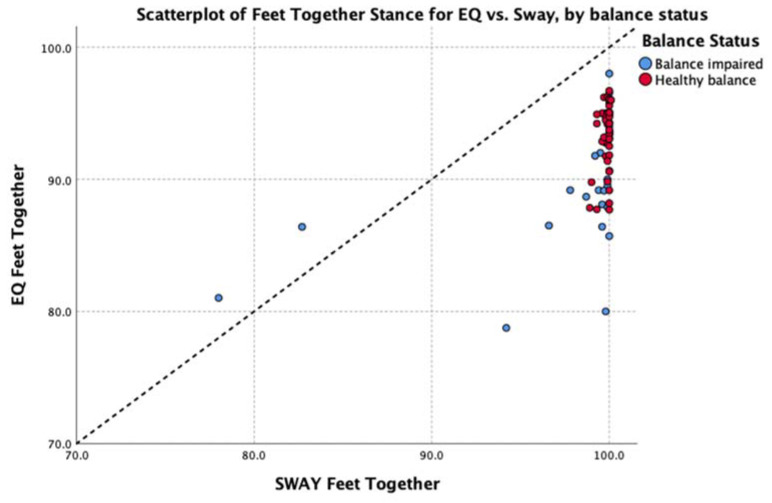
Scatterplot of feet together balance results in the healthy group.

The data were explored further with Spearman’s rho non-parametric test of correlation. This showed a strong, positive relationship between the average balance output (average of all five stances) of EQ Balance and Sway across the entire group (rho = 0.85, *p* < 0.01). Subgroup analysis showed a strong to moderate positive relationship for healthy and balance-impaired groups, respectively (healthy rho = 0.67, balance-impaired rho = 0.50, *p* < 0.01). Analysis of each individual stance found a significant positive correlation between the two measures on all stances except for the easiest pose (feet together) in the healthy group. Review of the scatterplot of this pose (Figure 6) indicates that this low correlation is due to a very low degree of variability in the results, which is to be expected in a healthy population performing an easy balance stance. Full details are shown in Table 4. 

The aim of this study was to evaluate the performance of EQ Balance, a gamified mobile balance assessment, and Sway, a more traditional mobile balance assessment, in the clinical setting. The specific objectives were to a) determine if EQ Balance results are substantially equivalent to the Sway results and b) evaluate the intrasession reliability, or test–retest reliability of the output of EQ Balance. In the context of this clinical study, test–retest reliability will be impacted by variability in the participants’ balance performance as well as measurement variability introduced by the device.

The analysis in support of the first objective consisted of a Deming regression. The Deming regression corroborated the correlation analysis, finding that average balance output of EQ Balance and Sway across the entire group was not significantly different from the equation EQ = Sway + 0. This supports equivalence of EQ Balance to Sway Balance for the average balance score across all five stances. This holds true across the range of performance values when healthy and balance-impaired groups are included. However, the Deming regression identified some poses/groups where the slope and intercept were significantly different from 1 and 0, respectively. This indicates that the results of individual poses should not be interpreted interchangeably between the two devices. Instead, these results support the use of the average of all five poses as the primary clinical output. That is, during a clinical balance assessment, no single pose should be assessed in isolation. This is consistent with instructions for use for EQ Balance, Sway (the predicate device), and the mBESS assessment that was used by the clinician to assess the concussed patients included in this study. It is important that device labelling clearly indicates this precaution. 

The analysis of the second objective found strong consistency of the task output between test sessions. Consistency of the EQ Balance measure was conducted by calculating the intra-class correlation (ICC) on two consecutive EQ Balance tests. The ICC for the cohort was 0.87 (*p* < 0.001).

While the mBESS test is a fast, reliable measure of balance, this digital assessment tool provides the potential for individuals to assess their balance independently and track performance over time. More importantly, such tools provide objective vs subjective measures of balance function and remove the potential effect of inter-rater variability on balance assessment and re-assessment.

### 4.1. Future Directions

The demonstration of repeated measurements of balance allows individuals to establish their personalized healthy balance results. By effectively comparing a new result against this individual’s history, as opposed to being compared to a heterogeneous normative population dataset, it may be possible to more accurately identify subtle deviations from an individual’s healthy range, thus enabling a more informed assessment of the significance and causality of change.

Future work should continue to characterize typical healthy values and additional factors that may influence EQ Balance results in healthy participants. For example, the effects of transient factors such as acute exercise and sleep, as well as personal differences including body type (e.g., height), history of injury, and physical literacy. 

In addition, future studies should examine the reliability of EQ Balance over varying number of timepoints as well as time between points to identify factors such as learning effects. 

Future studies should compare EQ Balance to the results of a force platform, the putative gold standard of balance assessment.

Finally, future work could focus on the clinical utility of EQ Balance in screening for, assessing, and monitoring specific conditions and diseases. The post-hoc analysis presented in the current study reveals a significant difference between healthy and impaired-balance groups. Figure 5 shows a cluster of results above 80, and a long tail of results below this value, mostly including balance-impaired patients. These results could provide pilot data for an investigation into a clinically-significant EQ Balance threshold that indicates balance impairment or increased risk of falls. To further substantiate such an investigation, it would be important to collect symptom severity and time since injury.

### 4.2. Limitations

The study sample was limited to 70 volunteers, which could limit the generalizability of the results. Theoretically, a larger sample size should lead to an increase in the strength of the observed relationships; however, one cannot know how the results will generalize to all populations. In addition, no effect of age or sex was observed; however, as this was not the focus of the study, it is likely that the sample size was underpowered to observe such an effect. Finally, it is important to recognize that EQ Balance is not intended to replicate the complexity of an appropriately credentialed clinician-mediated medical history and clinical neurological exam. 

## 5. Conclusions

EQ demonstrated safety and reliability in measuring balance function in healthy and concussed patient populations. The Deming regression showed strong agreement between average balance measures (the primary device output), and agreement on all poses except right single leg and tandem left. Test–retest reliability was shown by ICC analysis, which demonstrated strong consistency of the task output between test sessions. Poorer balance scores were observed for subjects with balance impairments, which included subjects with brain injury, suggesting potential for further research is recommended on the clinical utility of EQ Balance in screening for, assessing, and monitoring specific conditions and diseases, while the mobile application makes it enticing to study in many different settings. Overall, EQ Balance, as part of EQ Brain Performance, adds value to the existing balance assessment tools as an objective accurate balance measure presented in a novel, gamified way. 

## Figures and Tables

**Table 1:** Demographic descriptors of the cohort (*n* = 70).

Cohort	Entire Cohort	Healthy	Concussed	Balance-Impaired
Age, continuous (units: mean years ± standard deviation)	37.8 ± 14.8	35.6 ± 12.3	39.6 ± 16.4	45.6 ± 12.7
Age, categorical (units: number of participants)				
≤ 18 years	12	3	9	2
18–65 years	58	28	30	24
≥ 65 years	0	0	0	0
Sex Women Men	45 25	16 15	29 10	18 8

**Table 2:** Method comparison summary—Spearman’s correlation and Deming regression.

Grouping Variable	Spearman’s Rho		Coefficient of VAR.	Deming EquationEQ = B0 × Sway + B1	T-Stat (*p*-val) Intercept ≠ 0	T-stat (*p*-val) Slope ≠ 1
**Comparison by pose: all participants (*n* = 70)**							
Feet together	0.49	(*p* < 0.001)	EQ: 3.53 Sway: 2.01	y = 0.87x + 6.02	0.06 (0.95)	0.12 (0.90)
Tandem left	0.82	(*p* < 0.001)	EQ: 12.35 Sway: 17.80	y = 0.70x + 21.47	1.69 (0.09)	2.22 (0.03)*
Tandem right	0.78	(*p* < 0.001)	EQ: 13.06 Sway: 18.18	y = 0.84x + 8.12	0.30 (0.77)	0.54 (0.59)
Left single leg	0.78	(*p* < 0.001)	EQ: 16.62 Sway: 27.44	y = 0.74x + 21.89	1.85 (0.07)	1.69 (0.10)
Right single leg	0.79	(*p* < 0.001)	EQ: 17.73 Sway:27.53	y =0.47x + 42.50	6.19 (0.00)*	6.16 (0.00)*
Average of all poses	0.85	(*p* < 0.001)	EQ: 5.71 Sway: 9.02	y = 0.76x + 18.36	1.36 (0.18)	1.54 (0.13)
**Comparison by balance status (average score of all 5 poses)**							
healthy balance group (*n* = 44)	0.67	(*p* < 0.001)	EQ: 5.71 Sway: 9.02	y = 0.54x + 37.75	3.47 (0.00)*	3.87 (0.00)*
impaired balance group (*n* = 26)	0.50	(*p* < 0.01)	EQ: 5.71 Sway: 9.02	y = 0.80x + 15.46	0.45 (0.65)	0.43(0.67)
**Comparison by sex (average score of all 5 poses)**							
Women (*n* = 45)	0.80	(*p* < 0.001)	EQ: 5.71 Sway: 9.02	y = 0.44x + 47.64	3.23 (0.00)*	3.50(0.00)*
Men (*n* = 25)	0.86	(*p* < 0.001)	EQ: 5.71 Sway: 9.02	y = 0.71x + 21.15	0.40 (0.69)	0.38(0.71)
**Comparison by performance: split by Sway values above/below 80 (average score of all 5 poses)**							
Sway >= 80 (*n* = 47)	0.72	(*p* < 0.001)	EQ: 5.71 Sway: 9.02	y = 1.70x - 67.63	−2.00 (0.05)	1.87 (0.07)
Sway < 80 (*n* = 23)	0.54	(*p* < 0.01)	EQ: 5.71 Sway: 9.02	y = 0.99x + 6.71	0.12 (0.90)	−0.01 (0.99)

**Table 3:** Comparison of balance scores by group: healthy vs. impaired balance.

Pose	Healthy Balance (*n* = 44) Median (5% to 95% Range)	Impaired Balance (*n* = 26) Median (5% to 95% Range)	Mann-Whitney Z-Value (*p*-Value)
**EQ Balance scores**			
Feet together	94.0 (87.8 to 96.2)	89.4 (79.2 to 97.5)	−3.30 (*p* < 0.001)
Tandem left	91.4 (68.0 to 94.8)	73.3 (31.2 to 92.8)	−5.04 (*p* < 0.000)
Tandem right	90.0 (74.4 to 95.2)	75.5 (42.7 to 94.0)	−4.86 (*p* < 0.000)
Left single leg	83.2 (60.5 to 92.7)	61.0 (25.0 to 85.3)	−5.16 (*p* < 0.000)
Right single leg	81.6 (61.1 to 90.5)	57.5 (11.4 to 82.3)	−5.54 (*p* < 0.000)
Average 5 poses	87.0 (77.3 to 91.8)	73.5 (46.6 to 88.0)	−6.08 (*p* < 0.000)
**Sway Balance Scores**			
Feet together	100.0 (99.1 to 100.0)	99.8 (79.6 to 100.0)	−2.62 (*p* < 0.009)
Tandem left	96.5 (83.9 to 99.6)	79.0 (6.2 to 98.6)	−5.22 (*p* < 0.000)
Tandem right	98.0 (80.4 to 99.9)	82.9 (5.6 to 99.4)	−4.64 (*p* < 0.000)
Left single leg	84.5 (13.7 to 97.5)	61.4 (0.0 to 88.4)	−4.28 (*p* < 0.000)
Right single leg	84.1 (39.2 to 97.3)	59.3 (0.0 to 79.2)	−5.12 (*p* < 0.000)
Average 5 poses	90.4 (73.2 to 97.6)	67.7 (28.2 to 89.8)	−5.96 (*p* < 0.000)

**Table 4:** Variability of results for the feet together stance, separated by healthy vs. balance-impaired groups.

Group	N	Range	Minimum	Maximum	Mean	Std. Deviation
**Healthy**						
EQ Feet Together	44	9.0	87.7	96.7	93.3	2.52
SWAY Feet Together	44	1.2	98.9	100.1	99.8	0.28
**Balance-impaired**						
EQ Feet Together	26	19.3	78.8	98.0	89.6	4.88
SWAY Feet Together	26	22.0	78.0	100.0	97.9	5.35
